# Development of a Digital Research Assistant for the Management of Patients’ Enrollment in Oncology Clinical Trials within a Research Hospital

**DOI:** 10.3390/jpm11040244

**Published:** 2021-03-27

**Authors:** Alfredo Cesario, Irene Simone, Ida Paris, Luca Boldrini, Armando Orlandi, Gianluca Franceschini, Filippo Lococo, Emilio Bria, Stefano Magno, Antonino Mulè, Angela Santoro, Andrea Damiani, Daniele Bianchi, Daniele Picchi, Guido Rasi, Gennaro Daniele, Alessandra Fabi, Paolo Sergi, Giampaolo Tortora, Riccardo Masetti, Vincenzo Valentini, Marika D’Oria, Giovanni Scambia

**Affiliations:** 1 Open Innovation Unit, Scientific Directorate, Fondazione Policlinico Universitario A. Gemelli IRCCS, 00168 Roma, Italy; alfredo.cesario@policlinicogemelli.it (A.C.); irene.simone@guest.policlinicogemelli.it (I.S.); 2 Division of Gynecologic Oncology, Department of Woman and Child Health and Public Health, Fondazione Policlinico Universitario A. Gemelli IRCCS, 00168 Roma, Italy; ida.paris@policlinicogemelli.it (I.P.); giovanni.scambia@policlinicogemelli.it (G.S.); 3 Department of Imaging, Oncological Radiotherapy, and Hematology, Fondazione Policlinico Universitario A. Gemelli IRCCS, 00168 Roma, Italy; luca.boldrini@policlinicogemelli.it (L.B.); andrea.damiani@policlinicogemelli.it (A.D.); vincenzo.valentini@policlinicogemelli.it (V.V.); 4 Comprehensive Cancer Center, Fondazione Policlinico Universitario A. Gemelli IRCCS, 00168 Roma, Italy; armando.orlandi@policlinicogemelli.it (A.O.); emilio.bria@policlinicogemelli.it (E.B.); giampaolo.tortora@policlinicogemelli.it (G.T.); 5 Department of Woman and Child Health and Public Health, Fondazione Policlinico Universitario A. Gemelli IRCCS, 00168 Roma, Italy; gianluca.franceschini@policlinicogemelli.it (G.F.); antonino.mule@policlinicogemelli.it (A.M.); angela.santoro@policlinicogemelli.it (A.S.); riccardo.masetti@policlinicogemelli.it (R.M.); 6 Thoracic Surgery Unit, Fondazione Policlinico Universitario A. Gemelli IRCCS, 00168 Roma, Italy; filippo.lococo@policlinicogemelli.it; 7 Center for Integrative Oncology, Fondazione Policlinico Universitario A. Gemelli IRCCS, 00168 Roma, Italy; stefano.magno@policlinicogemelli.it; 8 Information and Communication Technology Unit, Fondazione Policlinico Universitario A. Gemelli IRCCS, 00168 Roma, Italy; daniele.bianchi@policlinicogemelli.it (D.B.); daniele.picchi@unikey.it (D.P.); paolo.sergi@policlinicogemelli.it (P.S.); 9 Unikey srl, 00168 Roma, Italy; 10 Clinical Trial Center S.p.A., 00168 Roma, Italy; guido.rasi@policlinicogemelli.it (G.R.); gennaro.daniele@policlinicogemelli.it (G.D.); 11 Phase I Unit, Fondazione Policlinico Universitario A. Gemelli IRCCS, 00168 Roma, Italy; 12 Precision Medicine in Senology Unit, Scientific Directorate, Fondazione Policlinico Universitario A. Gemelli IRCCS, 00168 Roma, Italy; alessandra.fabi@policlinicogemelli.it; 13 Dipartimento di Medicina e Chirurgia Traslazionale, Università Cattolica del Sacro Cuore, 00168 Roma, Italy; 14 Scientific Directorate, Fondazione Policlinico Universitario A. Gemelli IRCCS, 00168 Roma, Italy

**Keywords:** clinical trial, patient enrollment, artificial intelligence, machine learning, breast cancer, lung cancer, oncology, web app, personalized medicine

## Abstract

Clinical trials in cancer treatment are imperative in enhancing patients’ survival and quality of life outcomes. The lack of communication among professionals may produce a non-optimization of patients’ accrual in clinical trials. We developed a specific platform, called “Digital Research Assistant” (DRA), to report real-time every available clinical trial and support clinician. Healthcare professionals involved in breast cancer working group agreed nine minimal fields of interest to preliminarily classify the characteristics of patients’ records (including omic data, such as genomic mutations). A progressive web app (PWA) was developed to implement a cross-platform software that was scalable on several electronic devices to share the patients’ records and clinical trials. A specialist is able to use and populate the platform. An AI algorithm helps in the matchmaking between patient’s data and clinical trial’s inclusion criteria to personalize patient enrollment. At the same time, an easy configuration allows the application of the DRA in different oncology working groups (from breast cancer to lung cancer). The DRA might represent a valid research tool supporting clinicians and scientists, in order to optimize the enrollment of patients in clinical trials. User Experience and Technology The acceptance of participants using the DRA is topic of a future analysis.

## 1. Introduction

Cancer care is a complex pathway that is based on a multidisciplinary collaboration among professionals who share the latest evidence and pool their expertise and information through regular communication flows [1]. Multidisciplinary data sharing is an essential approach for tracing patients’ pathways, optimizing therapeutic opportunities, and improving healthcare quality. This approach increases evidence-based practice and avoids treating patients outside standardized protocols and recommended guidelines [2,3].

Clinical trials are imperative for testing novel cancer treatments, advancing the knowledge of care, and determining the best strategies to enhance patients’ survival and quality of life outcomes [4,5]. Nevertheless, the possible lack of communication and real-time synchronization among professionals may produce a fragmentation of services and practices, potentially resulting in the non-optimization of patients’ accrual in clinical trials and the limitation of their access to innovative therapeutic solutions [4,5,6].

One possible solution can be represented by data sharing approaches, facilitating the enrollment of patients in clinical trials that allow for increasing the chances of recovery, testing novel treatments, and improving knowledge of disease. Less than 5% of the patients are currently enrolled in clinical trials due to logistical issues, a lack of resources, and difficulty in data sharing [4,7,8,9].

Our research hospital has a notable oncological vocation, with nearly 60,000 patients annually accessing our facility with its complex organization in clinical, surgical, and service departments that welcome and manage all of the needs of the cancer patients. Specifically, the Comprehensive Cancer Center coordinates and optimizes all of the cancer related activities, guaranteeing the functionality of specific multidisciplinary working groups and the access to innovative therapies through enrollment in clinical trials or comprehensive interpretation of big data at the institutional and network levels [9,10,11]. In order to reduce daily communication inconveniences [12,13], a specific platform, called “Digital Research Assistant” (DRA), was developed to report real-time every available clinical trial active within our research hospital and assist clinicians in properly matching patients with the more appropriate studies.

The aim of this paper is to show how the DRA was implemented for breast cancer clinical trials to map all of the active studies on this specific disease and encouraging proper patients’ enrollment. Its scalability was also evaluated presenting the lung cancer case study.

## 2. Materials and Methods

### 2.1. Ideation

**Figure 1:**
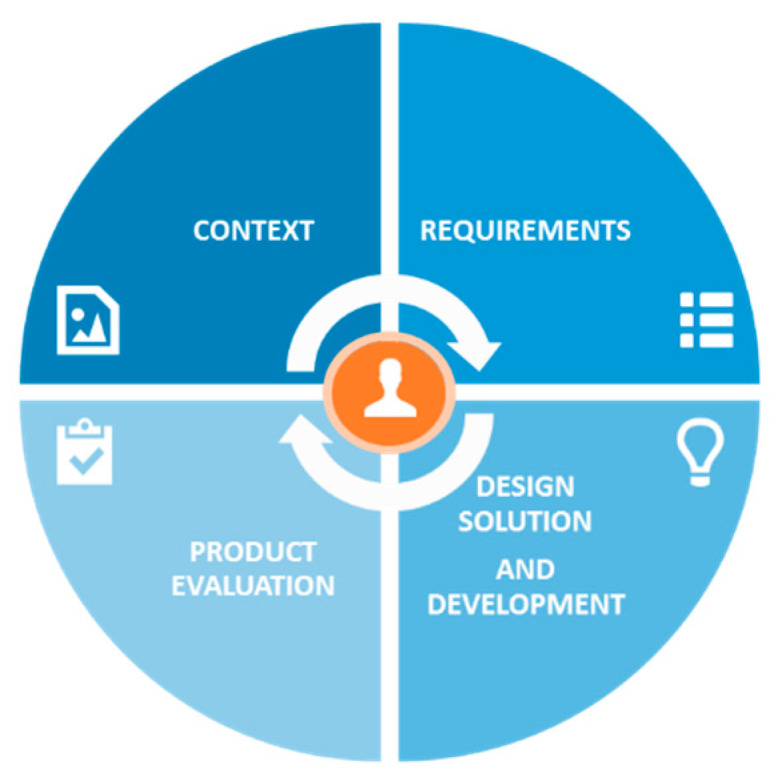
User-centered designed approach. Context: the program manager identifies who are the primary users of the product, how and why they will use it, what are their needs, and which environment they will use the tool. Requirements: when the context is defined, the program manager identifies the detailed requirements of the product, according to the needs of the user. Design solutions and development: once goals and requirements are settled, the ICT professionals and the project manager design and develop the tool for its usability. Evaluate Product: product designers (in this case, ICT professionals) run usability tests to obtain users’ feedback on the product.

A project manager and two Information and Communication Technology (ICT) professionals started a pilot project with the Breast and Lung Cancer institutional Working Groups, following a user-centered designed approach [14] (Figure 1).

Healthcare professionals of the involved working groups agreed on nine minimal fields of interest to preliminarily classify the characteristics of patients’ records in the platform (Table 1) and obtain a quick evaluation of the patients and its possible link to the active and open clinical trials, using breast cancer as a case study.

TNM classification and corresponding stage were obtained through the input of numerical values according to the 8th edition of TNM classification of malignant tumor [15];

- age is a continuous numerical value;
- immunophenotype consists on the classification into 4 subtypes of breast cancer according to the cellular expression of estrogen (ER: positive or negative) and progesterone (PgR: positive or negative) receptors, epidermal growth factor receptor 2 (HER2: positive or negative) and the proliferation index (Ki67: from 1% to 100%):
- Luminal A: ER positive and/or PgR positive, HER2 negative, Ki 67 < 25%;
- Luminal B: ER positive and/or PgR positive, HER2 negative, Ki 67 > 25%;
- HER2 positive: any expression of ER, PgR and Ki67, but HER2 positive;
- triple negative: negativity of ER, PgR and HER2 and any expression of Ki67;
- histological examination allows to acquire the information of whether the tumor tissue sample is available at our institution or not (internal or external);
- BMI is calculated automatically, underlining when value is greater than 25 which represents a general risk factor;
- the stage of therapy indicates the type of systemic treatment that the patient is undergoing: neoadjuvant, adjuvant, first line, and beyond the first line in metastatic setting;
- genetic test indicates patient’s BRCA1/BRCA2 or multigenic panel mutational status; and,
- PI3Kmutation indicates the mutational status of this specific gene.

Particularly, prognosis and treatment are determined by the stage (TNM classification) of the tumor at the time of diagnosis, but also by the histological/molecular subtype that is obtained with biopsies or in the definitive pathological examination.

### 2.2. Implementation

The DRA was created with the aim to meet several essential clinical and research points:

- define an operational app that allows to update data informing all users in real-time;
- ensure GDPR-compliant data security;
- allow access to both authorized internal and external users (i.e., for multicentric studies);
- implement a scalable infrastructure manageable by various specialists (i.e., medical doctors, data managers, research nurses, etc.); and,
- develop a matchmaking algorithm between eligible patients and clinical trials.

**Figure 2:**
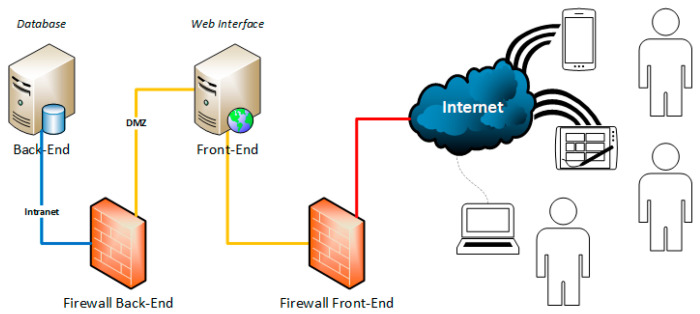
Hardware infrastructure.

The infrastructure was designed and developed by separating the front-end (i.e., the exposed services) from the back-end (information content) in order to ensure data protection and security (Figure 2).

Infrastructure versatility was then tested using a different case study (lung cancer), to confirm the possibility to easily adapt the platform for indications other than the one used for the first development.

#### 2.2.1. Technologies and Software

A progressive web app (PWA) was developed to implement a cross-platform software scalable on several electronic devices (i.e., PC, tablet, smartphone). Differently from classic web apps, a PWA that is installed on mobile devices acts as if it was a native app of the device itself, allowing:

- to use its functionalities with all the browser through a reference URL, without installation;
- to adapt the display according to the screen size of the device; and,
- to access its functionalities off-line, guaranteeing data loading by using micro service technologies (APIs).

Business logic was developed using the Microsoft, NetCore 3.1 Framework. This software was structured with APIs that make access to data in secure mode with a https protocol scalable and decoupled from the front-end. The app is scalable in terms of the evolution and reutilization of the code, as well as maximization of loading information on the network.

The angular open source framework version 9.07 was used to develop the front-end, directly running from the browser after being downloaded from the web server. This choice was taken to have an advance in terms of efficiency, saving the exchange of information between client and server every time that there is a request for action by the user.

The SignalR open source framework was used to guarantee a real-time update of data, even when the app is open on a browser. This technology automatically updates information modified by other users, using a two-way channel between the client (browser) and the server (web app). In order to ensure the communication of changes in information to clients, even when they are not connected to the web app, neither it is open, push notifications have been activated using the Google Firebase engine. This open source service allows for sending messages through a web service that transmits notifications to the users of the service. Finally, Microsoft Sql Server 2016 Enterprise Edition was the DBMS used to define the relational model related to this architecture.

#### 2.2.2. Accessibility

**Figure 3:**
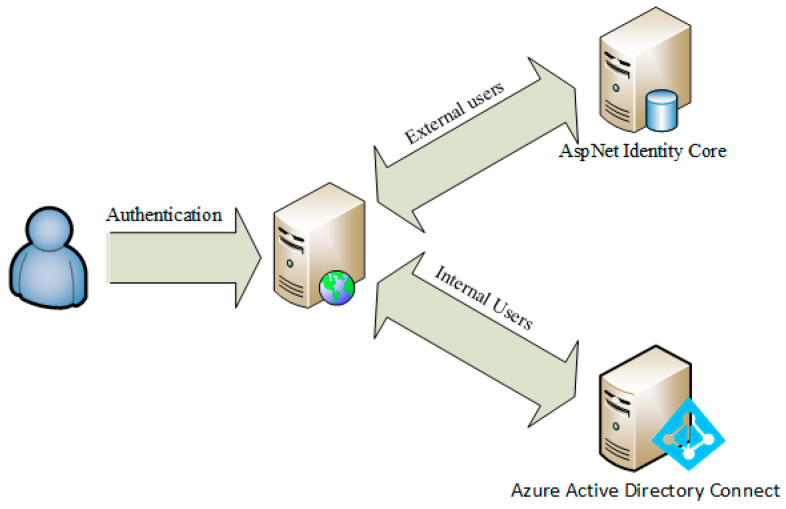
Authentication architecture.

System access is possible through a hybrid authentication architecture (Figure 3) that allows specialists and healthcare professionals located in various research centers to use the platform:

- internal users, through access with personal domain credentials; and,
- external users from other research centers (after compiling a standard registration form), through authentication managed internally by the application.

The app admits three profiles:

- System Administrator: enabled to manage configuration features of the app, as described in the “Functionalities” section;
- User: enabled to input information about a patient, to enroll and to ask for patients enrollment; and,
- Study Manager: enabled to use the same functions of the “User” profile, as well as to manage the creation and modification of trials.

“System Administrators” manage the access of internal users (with “User” and “Study Manager” profiles) enabling them to use the app. An HR representative of the research hospital supervises the list of users.

## 3. Results

### 3.1. Functionalities and Configuration

From the side menu, the following functionalities are available:

- List of Patients
- List of Clinical Trials
- My Requests
- My Interests
- My Clinical Trials (or Studies)
- Pending Requests
- Configuration
  ○ Users
  ○ Clinical Trial (or Study)
  ○ Type of Clinical Trial (or Study)
  ○ Phase
  ○ Settings
- Enable notifications

Other functionalities include system management and configurations, which are dedicated to “System Administrator” profiles.

#### 3.1.1. Patients’ Management and Enrollment (Matching) to Clinical Trials

**Figure 4:**
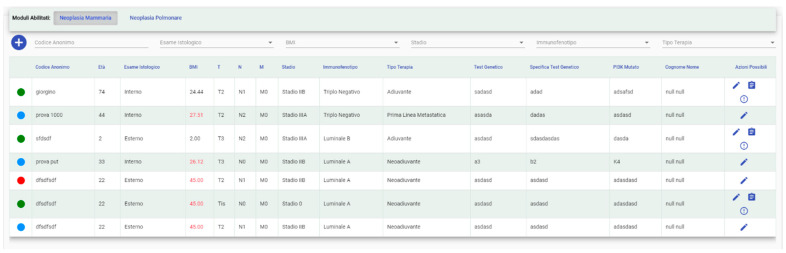
List of patients uploaded in the system (in Italian). Names are examples and do not correspond to real cases.

Under the operational functions, it is possible to see a real-time updated patients list from the activated module to:

- check their status with a color legend (enrolled, pending, etc.) (Figure 4);
- select the relevant specialist if the user has access to more than one;
- add a new patient and/or modify data related to a specific one;
- add a patient to a study (for “Study Manager” profiles) or send a request to the Study Manager of the selected trial to insert him (for “User” profiles);
- add a patient in your interests; and,

Search filters are available for a better user experience.

In particular, the legend includes four entries:

- Free (green): the patient can be enrolled in a trial;
- Enrolled (red): the patient is currently enrolled in a trial;
- Requested Enrollment (yellow): the patient has already been requested for a trial. The “Study Manager” will be able to deny the request or allow the patient in the study; and,
- Selected for Possible Enrollment: the patient has been selected from a “User” to be evaluated for possible enrollment.

Patient enrollment changes according to the logged profile. If the profile is “Study Manager”, then the enrollment occurs immediately, otherwise a “User” sends a request to the “Study Manager” of the selected trial, which allows or denies access to the patient in the study. When a patient is accepted, or directly recruited, the “Study Manager” inserts the starting and ending date of the trial.

#### 3.1.2. Clinical Trial Configuration

**Figure 5:**
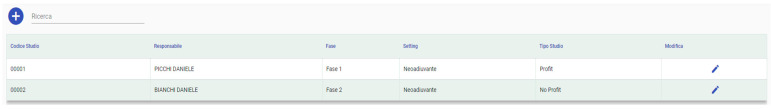
List of Clinical Trials (in Italian). Names are examples and do not correspond to real cases.

**Figure 6:**
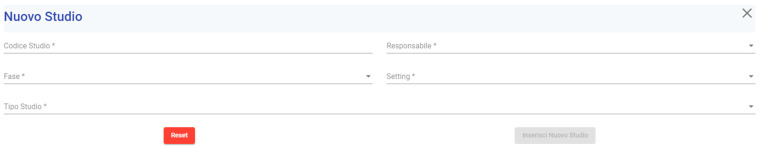
New Clinical Trial form (in Italian).

A list of Clinical Trials with their status (i.e., active, suspended, closed) is displayed for all of the profiles (Figure 5). To configure a Clinical Trial, the “User”, or the “Study Manager” can enter the information related to the study in which patients can be enrolled (Figure 6). These information are shared with other users, especially those that are interested in the same pathology.

#### 3.1.3. Phase of the Clinical Trial Configuration

**Figure 7:**
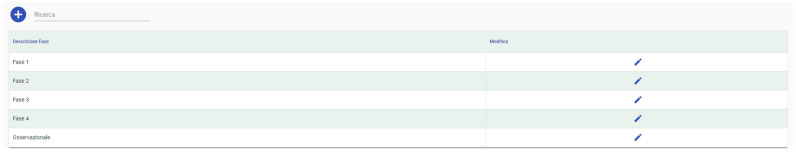
List of Phases (in Italian).

**Figure 8:**
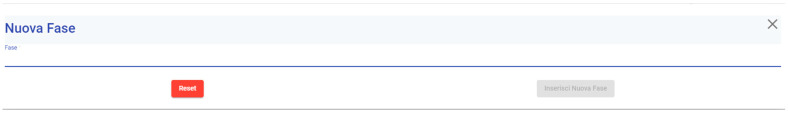
New Phase insert form (in Italian).

“User” and “Study Manager” profiles can input and modify the Phase of the clinical trial, visible on the selection menu while configuring a trial (Figure 7 and Figure 8).

Matchmaking option. An algorithm then configures the clinical trial. By defining the inclusion-exclusion criteria of a patient in a trial enrollment, these criteria become the rules of the algorithm that allows matchmaking between an eligible patient and a trial. When the “User” inserts a new patient, it is possible to click on the action “assign the patient to a study”. This action shows the list of clinical trials for which the patient is eligible. If the patient has characteristics that are coherent with the study, he/she can be enrolled. In particular, omic characteristics (such as genomic mutations) may help achieve a Personalized Medicine approach in oncological clinical trial enrollment.

As an enrichment of the services offered by the platform, a connection with the GEmelli NEtwoRk for Analysis and Tests in Oncology and medical Research “Generator”—Real World Data facility is offered to the clinician. Gemelli Generator Real-World Data is a research facility whose aim is the integration of the vast amount of patient data that are available in the Gemelli Data warehouse (about 700 million data items as measured at the end of December 2020). The generator takes care of the integration of these data items, in anonymized form, into specific datamarts, based on appropriate terminological systems, quality-checked and normalized with regard to the information originated from different, heterogeneous data sources, like traditional electronic health records (EHRs), omics data, Patient-Reported Experience Measures (PREMS), and Patient-Reported Outcome Measures (PROMS).

Machine Learning and Artificial Intelligence-based methods are at the heart of the Generator infrastructure, allowing for researchers to develop state of the art models, clustering, and decision support systems [16]. After the patient selection phase of the DRA, a simple user interface will give clinicians the opportunity to query the Generator datamarts for the availability of further covariates, referred to the selected patients, that can add more information to what is already present in the DRA core. Full integration between the two systems, at the ICT level, will guarantee an automatic and swift response in a privacy protected environment.

In this way, researchers can have a deeper view of the available data and formulate more study hypotheses, based on the large variety of information coming from heterogeneous data sources.

#### 3.1.4. Settings Configuration

**Figure 9:**
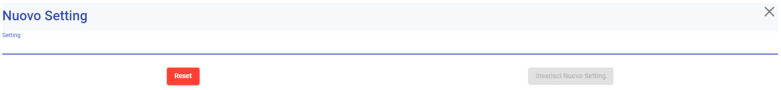
New Setting form (in Italian).

**Figure 10:**
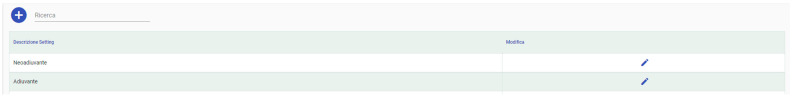
List of Settings descriptions (in Italian).

“System Administrators” can insert or modify the characteristics of the setting attributes (that are chosen by the WG) related to the clinical trials (Figure 9 and Figure 10), while “User” and “Study Manager” profiles can select them directly.

#### 3.1.5. User Requests

**Figure 11:**
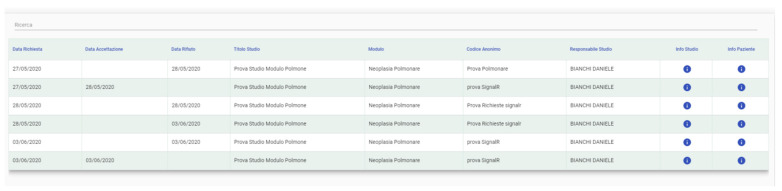
User Requests (in Italian). Names are examples and do not correspond to real cases.

In the “User Requests” section, all of the requests and their status (accepted, refused, and pending for evaluation) are displayed as well as other users’ information requests about a patient or a trial (Figure 11).

#### 3.1.6. Requests Management

**Figure 12:**
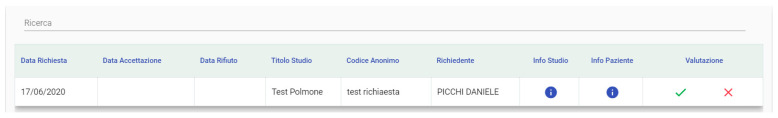
Requests Management (in Italian). Names are examples and do not correspond to real cases.

This section is only accessible to “Study Manager” profiles and allows accepting or declining a request (Figure 12). Each row shows a single request with the possibility of examining patient or trial information.

#### 3.1.7. Clinical Trials List

**Figure 13:**
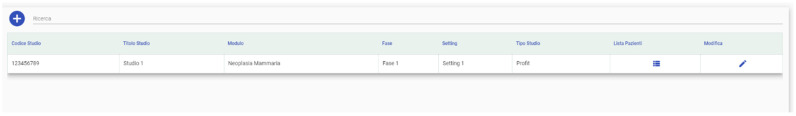
Trials list (in Italian). Names are examples and do not correspond to real cases.

This section shows a list of all the clinical trials that the logged profile is responsible for. The “Study Manager” profile also allows editing information about the trials and examining the enrolled patients’ full list (Figure 13).

#### 3.1.8. Possible Enrollment List

**Figure 14:**
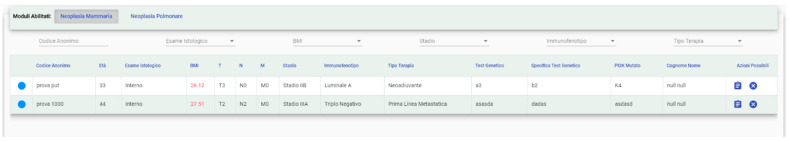
Possible enrollment list (in Italian). Names are examples and do not correspond to real cases.

This section shows all of the patients preferred by “User” profiles, and preference can be deselected. It is possible to send or accept requests of admission in a trial (Figure 14).

## 4. Customization

In this paper, we described the scale-up customization of the first DRA model on breast cancer to lung cancer thought for a high volume cancer care center. Table 2 shows all the varied characteristics of the patient, except for the “Age” field.

The parameters included in the “Minimum Fields” (Table 2) were selected while considering the main characteristics of lung cancer patients that can guide the accrual in clinical trials.

- TNM descriptors and the relative stage have been reported according to the criteria illustrated in the 8th edition of TNM classification of lung malignant tumor [17], where the clinical stage is determined according to radiological or radiometabolic assessment, while pathological staging is determined on the basis of pathological confirmations;
- age is a continuous numerical value;
- performance status was described according to the Eastern Cooperative Oncology Group (ECOG) criteria [18];
- surgery is considered only if performed with curative intent:
- histology: we indicated the most common histological subtypes among lung tumors;
- grading has been reported according to 2015 WHO Classification [19];
- resection margin status, indicating the pathological report of the specimen margin;
- genetic test indicates whether the patient has carried out genetic testing for ALK and ROS1 genes, EGFR and KRAS gene mutation and PDL1 expression; and,
- therapy type indicates which strategy of care has been adopted.

Prognosis and treatment are determined by disease stage (TNM classification), as identified by preliminary diagnostic investigations and histology. Surgery remains the main prognostic factor in the early-stage tumors and the completeness of resection (negative margin status) is a widely recognized factor influencing the long-term results in this setting. Otherwise, in locally advanced and metastatic stages, the molecular characterization represents the main determinant of long-term outcomes, based on the dramatic predictive role of featured biomarkers of activity/efficacy for molecular targeted agents and immunotherapy.

Table 3 shows the numerical data from a pilot test of the DRA database.

## 5. Discussion and Conclusions

Our solution exploited communication among professionals that are involved in oncological care and cancer research. The DRA we developed allows them to know all of the running clinical trials, guaranteeing all patients the best access to cure and research protocols, reducing the fragmentation of patients’ access to the oncological care-path with the multiple therapeutic intersections available in high volume centers (radiotherapy, surgery, and new lines of systemic therapy managed by multiple specialists).

This digital tool appeared to be well performing for patients’ data sharing within the single institution, but also in setting up networks with other cancer centers facilitating patients’ enrollment also for peripheral centers. In fact, in high-patient volume centers, such as our institution, the DRA seems a possible efficient resource to face this issue [20,21,22,23,24,25,26]. At the same time, the platform is easily moldable to the needs of different oncology work groups, as evidenced by the easy customization starting from the model for breast cancer to arrive at that for lung cancer.

Taking these considerations together, the platform might represent a valid research tool supporting clinicians and scientists, working in both high- and low-volume centers and the enrollment success rates for each matchmaking run is currently the object of in depth analysis and it will be a topic of future publications. User Experience and Technology Acceptance of participants using the DRA is topic of a second dedicated analysis.

## Figures and Tables

**Table 1:** Fields chosen by the professionals of the Breast Cancer Working Group, in order to classify the patients inserted in the platform.

Field	Value Type	Values	Notes
TNM	Text	T (1,2,3,4, IS) N (0,1,2,3) M (0,1)	
TNM stage	Numerical	From 0 to 4	If 1,2,3 specify the TNM
Age	Numerical	Range	
Immunophenotype	Text	Luminal A Luminal B Triple Negative HER 2 +	
Histological examination	Bit	Internal External	
BMI	Numerical	Mathematic formula	Specify if ≥25
Therapy stage	Text	Neoadjuvant Adjuvant First line metastatic After the first line	
Genetic test	Ternary	Positive Negative Not applicable	Possibility to specify the test
Mutated PI3K	Ternary	Yes No Not applicable	

**Table 2:** Fields chosen by the professionals of the Lung Cancer Working Group, in order to classify patients inserted in the platform.

Field	Value Type	Values	Notes
Patient Code (Social Security Number)	Text	Alphanumeric	
Pathological TNM	Text	pT (X, 0, 1a, 1b, 1c, 2a, 2b, 3, 4) pN (X, 0, 1, 2, 3) pM (X, 0, 1a, 1b, 1c)	Only for complete oncological interventions Information not mandatory, only if available
Clinical TNM descriptors	Numerical	cT (X; 0; 1a; 1b; 1c; 2a; 2b; 3; 4) cN (X; 0; 1; 2; 3) cM (X; 0; 1a; 1b; 1c)	
Clinical Stage	Numerical	Occult, 0, IA1, IA2, IA3, IB, IIA, IIB, IIIA, IIIB, IIIC, IVA, IVB	
Age	Numerical	Range	
ECOG performance status	Numerical	0; 1; 2; 3; 4	
Surgery	Binary	Yes; No	
Histology	Text	small cells carcinoma; adenocarcinoma; squamous cell carcinoma; other	
Grading	Text	G1; G2; G3	If applicable
Residual disease	Text	R0; R1	
Molecular characteristics	Text	Re-arrangement of ALK and ROS genes; EGFR and KRAS gene mutation; PDL1 expression	Information not mandatory, only if available
Therapy type	Text	Surgery; Chemotherapy; Immunotherapy; Radiotherapy; Other	

**Table 3:** Number of patients available in the Digital Research Assistant.

Pathology	N. Patients in the Database	N. Patients Requested for a Trial	N. Enrolled Patients
Breast Cancer	62	1	0
Lung Cancer	34	6	0

## Data Availability

Data sharing not applicable.
